# Supplementary material for: NAD+ dependent UPRmt activation underlies intestinal aging caused by mitochondrial DNA mutations
Source: Nat Commun. 2024 Jan 16;15:546. doi: 10.1038/s41467-024-44808-z (PMC10791663; doi:10.1038/s41467-024-44808-z)
Supplement: Supplementary file 1 — Supplementary Information [file 41467_2024_44808_MOESM1_ESM.docx]

**Supplementary Information**

**NAD^+^ dependent UPR^mt^ activation underlies intestinal aging caused by mitochondrial DNA mutations**

Liang Yang^1,2,3†^, Zifeng Ruan^1,3,4†^, Xiaobing Lin^1,3†^, Hao Wang^1,3^,Yanmin Xin^1,3^, Haite Tang^1,3^, Zhijuan Hu^1,3,4^, Yunhao Zhou^1,3,4^, Yi Wu^1,2,3^, Junwei Wang^1,3^, Dajiang Qin^2,5^, Gang Lu^6^, Kerry M. Loomes^7^, Wai-Yee Chan^6^, Xingguo Liu^1,2,3^*

^1^ CAS Key Laboratory of Regenerative Biology, Joint School of Life Sciences, Guangzhou Institutes of Biomedicine and Health, Chinese Academy of Sciences; Guangzhou Medical University, Guangzhou, China.

^2^ Centre for Regenerative Medicine and Health, Hong Kong Institute of Science & Innovation, Chinese Academy of Sciences, Hong Kong SAR, China.

^3^ Guangdong Provincial Key Laboratory of Stem Cell and Regenerative Medicine, China-New Zealand Joint Laboratory on Biomedicine and Health, CUHK-GIBH Joint Research Laboratory on Stem Cells and Regenerative Medicine, Institute for Stem Cell and Regeneration, Guangzhou Institutes of Biomedicine and Health, Chinese Academy of Sciences, Guangzhou, China.

^4^ University of Chinese Academy of Sciences, Beijing, China.

^5^ Key Laboratory of Biological Targeting Diagnosis, Therapy and Rehabilitation of Guangdong Higher Education Institutes, The Fifth Affiliated Hospital of Guangzhou Medical University, Guangzhou, Guangdong, China.

^6^ CUHK-GIBH Joint Research Laboratory on Stem Cells and Regenerative Medicine, CUHK-Jinan University Key Laboratory for Regenerative Medicine, Ministry of Education, School of Biomedical Sciences, Faculty of Medicine, The Chinese University of Hong Kong, Hong Kong SAR, China.

^7^ School of Biological Sciences and Institute for Innovation in Biotechnology, University of Auckland, Auckland 1010, New Zealand.

* Correspondence: [liu_xingguo@gibh.ac.cn](mailto:liu_xingguo@gibh.ac.cn)

†These authors contributed equally to this work

**Supplementary Figures 1-10**

**
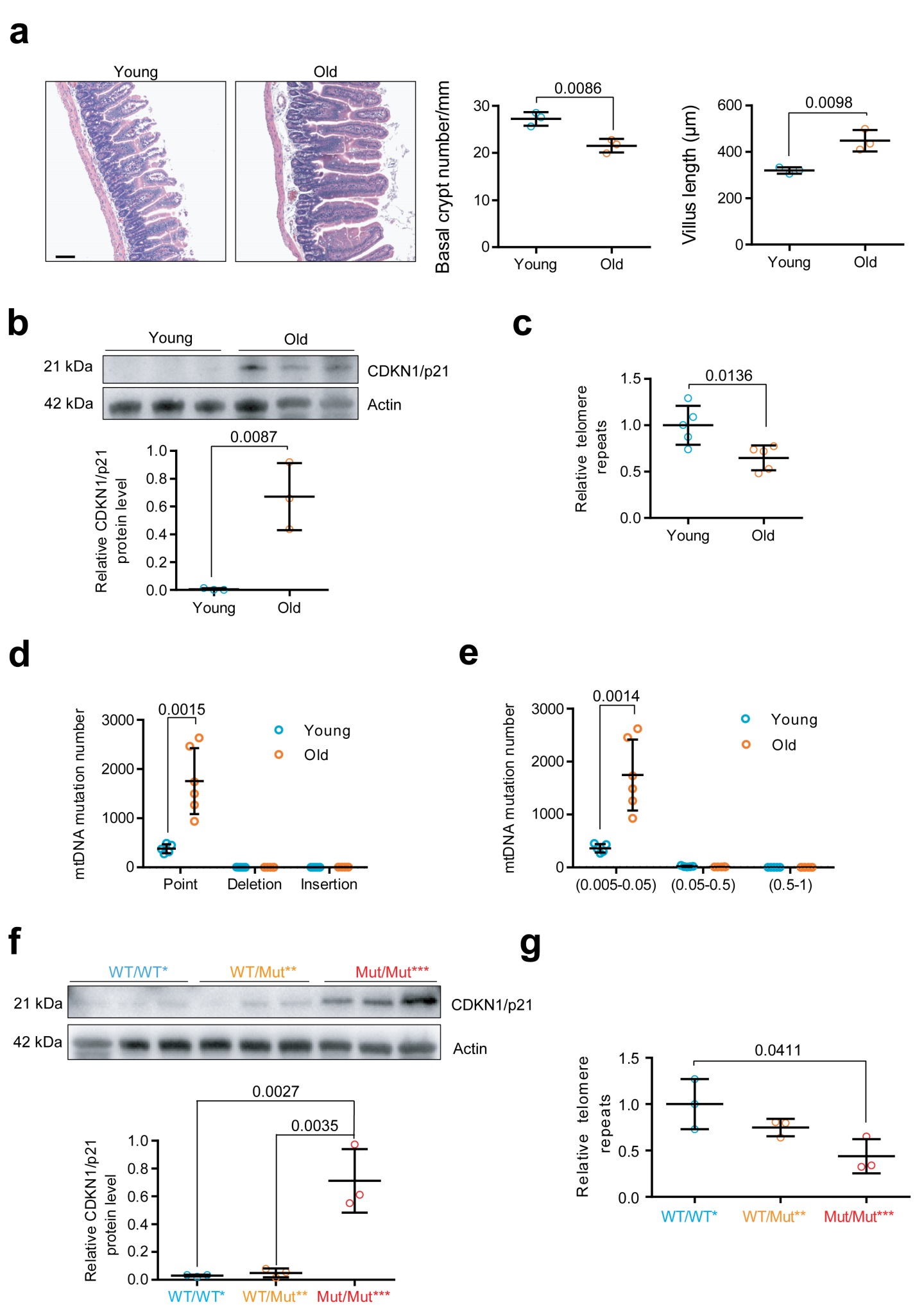
**

**Supplementary Fig. 1 Increased mtDNA mutation burden induces an aging-like phenotype in the small intestine, related to** **Fig. 1. a** Representative images of small intestine stained with H&E in young (3 months) and old (20 months) mice (Scale bar, 100 μm). Villus length and number of crypts per millimeter of small intestine are quantified on right (Data are presented as the mean ± S.D and n=3 mice per group; unpaired two-tailed Student’s t test). **b** Protein expression of CDKN1/p21 by western blot analysis in the small intestine of young (3 months) and old (20 months) mice. Relative band densities quantified using ImageJ are shown at bottom (Data are presented as the mean ± S.D and n=3 mice per group; unpaired two-tailed Student’s t test). **c** Relative telomere repeats by qPCR in the small intestine of young (3 months) and old (20 months) mice (Data are presented as the mean ± S.D and n=5 mice per group; unpaired two-tailed Student’s t test). **d** Number of mtDNA point mutations, mtDNA deletions and mtDNA insertions in the small intestine of young (3 months) and old (20 months) mice (Data are presented as the mean ± S.D; n=5 mice for young group, n=6 mice for old group; unpaired two-tailed Student’s t test). **e** mtDNA point mutations binned by frequency in the ranges (0.005-0.05), (0.05-0.05) and (0.5-1) in panel **c** (unpaired two-tailed Student’s t test). **f** Protein expression of CDKN1/p21 by western blot analysis in the small intestine in WT/WT*, WT/Mut** and Mut/Mut*** mice at 8 months of age. Relative band densities quantified using ImageJ are shown at bottom (Data are presented as the mean ± S.D and n=3 mice per group; one-way ANOVA test). **g** Relative telomere repeats by qPCR in the small intestines of WT/WT*, WT/Mut** and Mut/Mut*** mice at 8 months of age (Data are presented as the mean ± S.D and n=3 mice per group; one-way ANOVA test). Source data are provided with this paper.


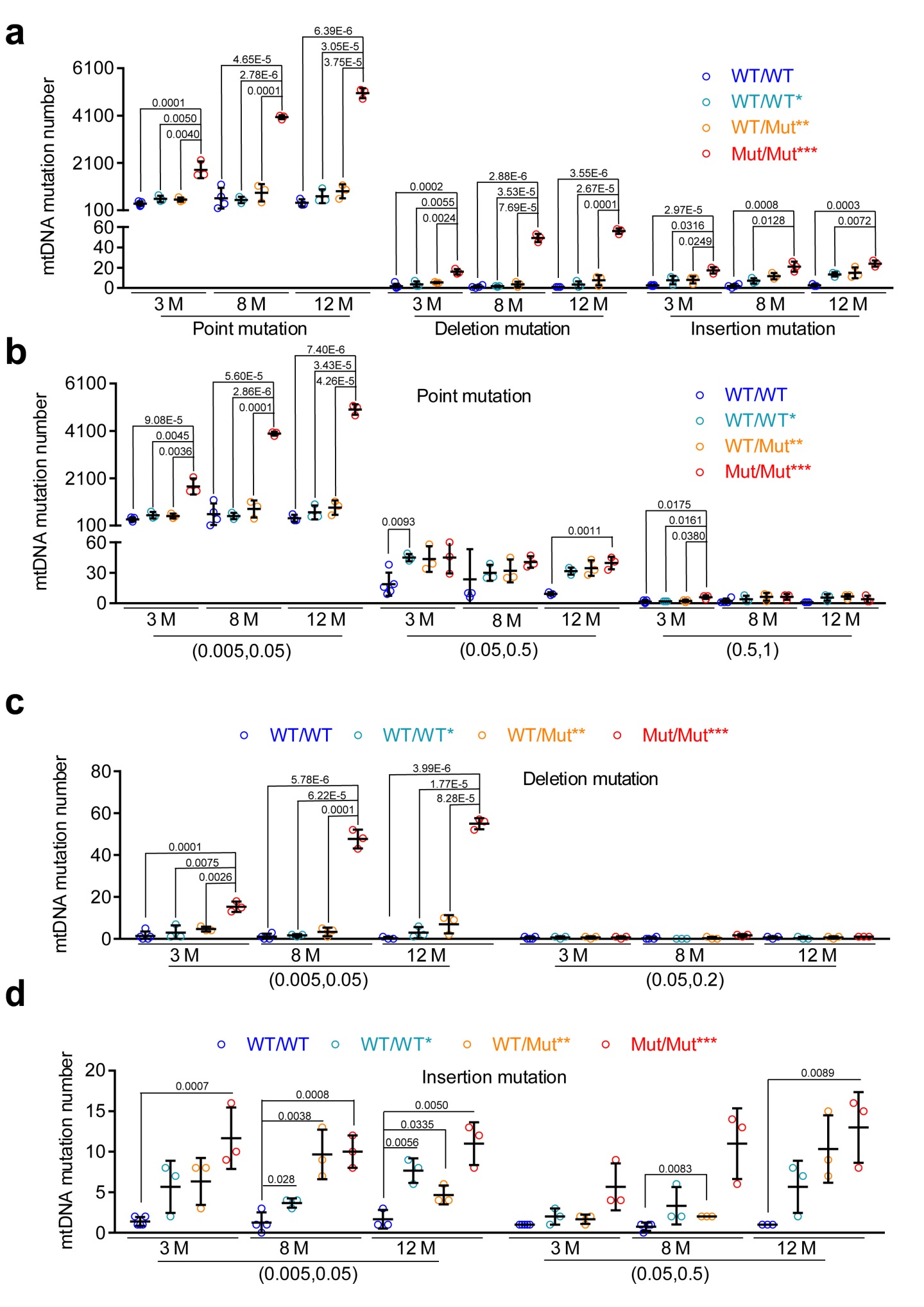


**Supplementary Fig. 2 mtDNA mutation accumulation in the small intestine during aging, related to** **Fig. 1. a** Number of mtDNA point mutations, mtDNA deletions and mtDNA insertions in WT/WT*, WT/Mut** and Mut/Mut*** mice at 3, 8 and 12 months of age (Data are presented as the mean ± S.D and n≥3 mice for each group; two-way ANOVA test). **b** mtDNA point mutations binned by frequency in the ranges (0.005-0.05), (0.05-0.05) and (0.5-1) in panel **a** (two-way ANOVA test). **c** mtDNA deletion mutations binned by frequency in the ranges (0.005-0.05) and (0.05-0.2) in panel **a** (two-way ANOVA test). **d** mtDNA insertion mutations binned by frequency in the ranges (0.005-0.05) and (0.05-0.05) in panel **a** (two-way ANOVA test). Source data are provided with this paper.


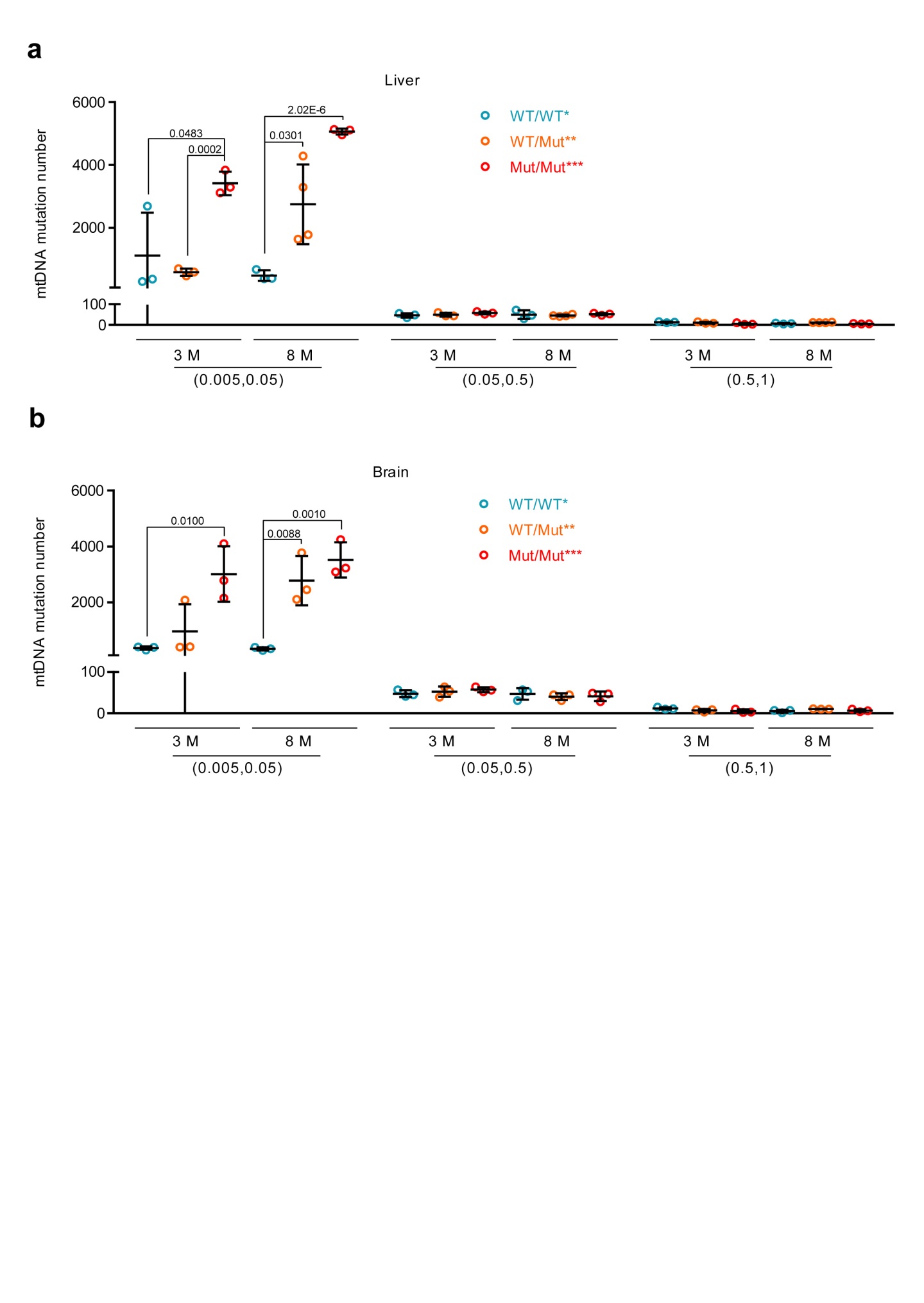


**Supplementary Fig. 3 mtDNA mutation accumulation in the liver and brain during aging, related to** **Fig. 1. a** mtDNA point mutations binned by frequency in the ranges (0.005-0.05), (0.05-0.05) and (0.5-1) in the liver of WT/WT*, WT/Mut** and Mut/Mut*** mice at 3 and 8 months of age (Data are presented as the mean ± S.D and n≥3 mice for each group; two-way ANOVA test). **b** mtDNA point mutations binned by frequency in the ranges (0.005-0.05), (0.05-0.05) and (0.5-1) in the brain of WT/WT*, WT/Mut** and Mut/Mut*** mice at 3 and 8 months of age (Data are presented as the mean ± S.D and n≥3 mice for each group; two-way ANOVA test). Source data are provided with this paper

**
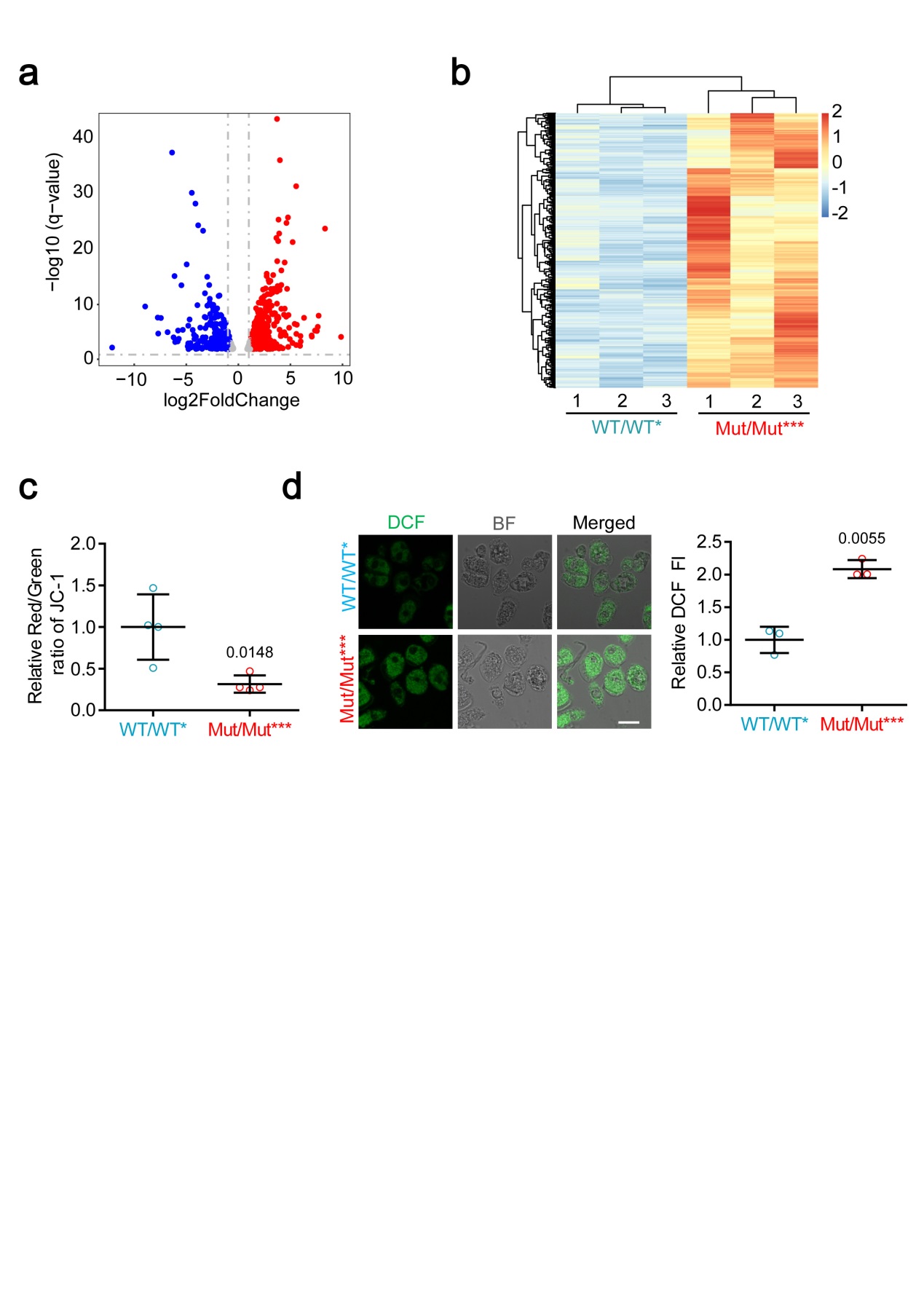
**

**Supplementary Fig. 4 Increased mtDNA mutation burden induces an intestinal aging phenotype by NAD^+^ depletion, related to** **Fig. 2**. **a** Volcano plot of differential genes in the small intestine of Mut/Mut*** mice at 8 months of age compared to WT/WT* mice (n=3 mice per group). 780 upregulated and 500 downregulated genes were identified using DESeq2, with p-adjust < 0.05 & abs (log2FoldChange)>1. **b** The heatmap of 780 upregulated differential genes in the intestine of Mut/Mut*** mice derived from panel **a**. **c** Relative Red (J-aggregates)/Green(J-monomers) ratio of JC-1 in the intestinal crypts of Mut/Mut*** mice and WT/WT* mice at 8 months using flow cytometry; the ratio is positively correlated with mitochondrial inner membrane potential (Data are presented as the mean ± S.D and n=4 mice per group; unpaired two-tailed Student’s t test). **d** Detection of ROS in the intestinal crypts of Mut/Mut*** mice and WT/WT* mice at 8 months using DCF imaging (Scale bar, 10 μm), and relative DCF FI value are quantified on right (Data are presented as the mean ± S.D and n=3 mice per group; unpaired two-tailed Student’s t test). Source data are provided with this paper.
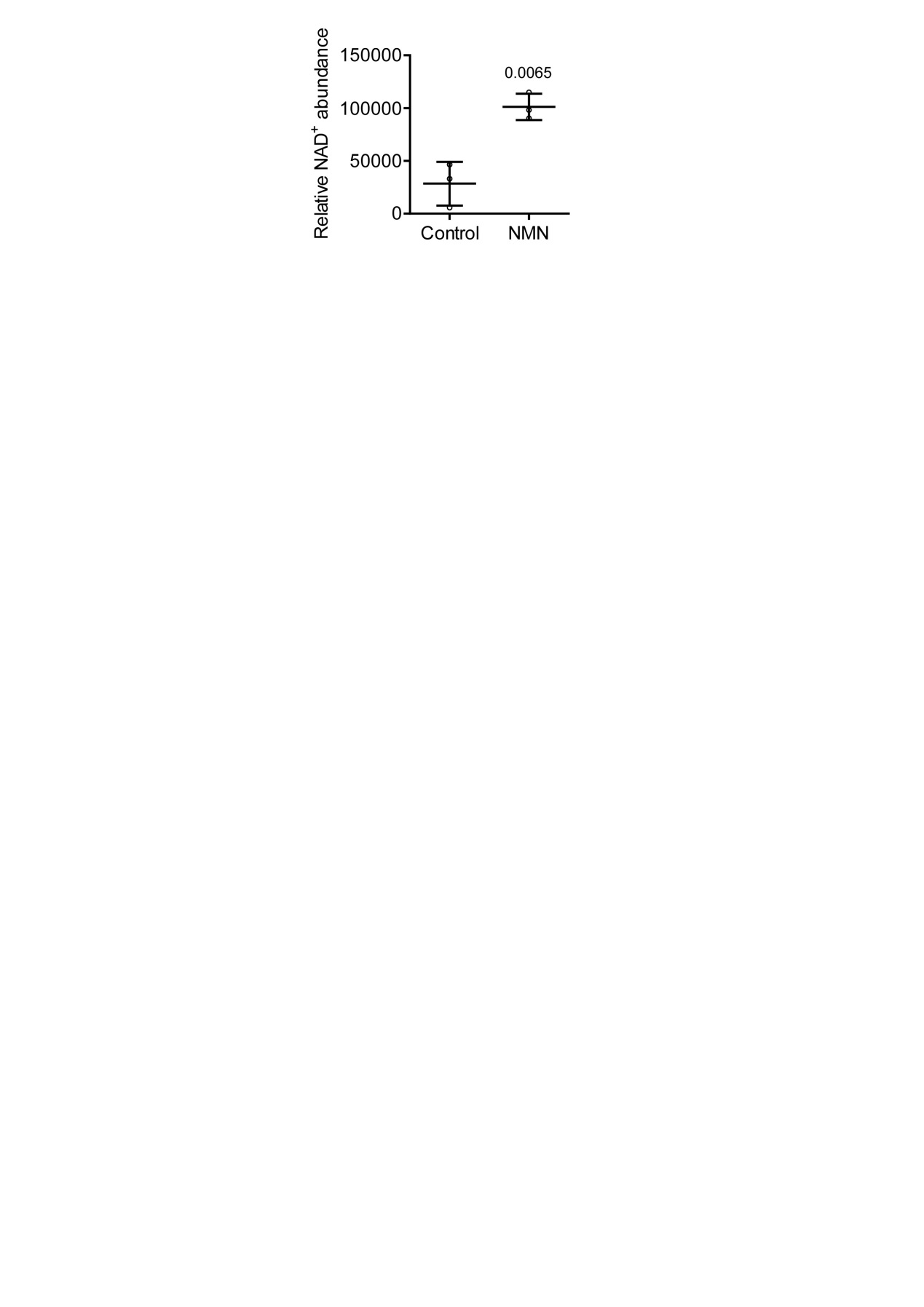
**Supplementary Fig. 5** Relative NAD^+^ abundance by mass spectrometry in the intestinal crypts of Mut/Mut*** mice at 8 months of age with NMN or water control (Data are presented as the mean ± S.D and n=3 mice per group; unpaired two-tailed Student’s t test), **related to** **Fig. 3**. Source data are provided with this paper.

**
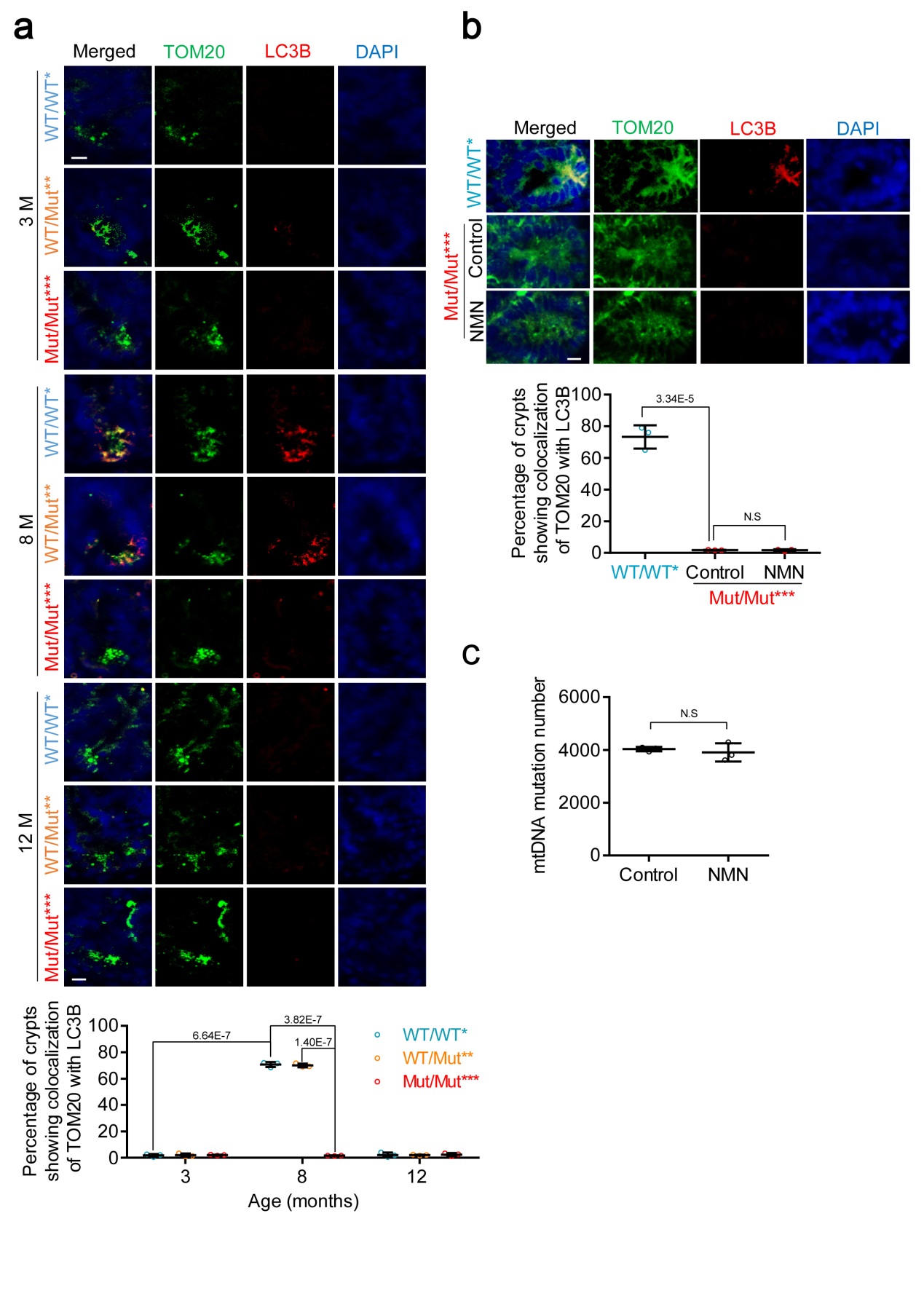
**

**Supplementary Fig. 6 Intestinal aging phenotype induced by NAD^+^ depletion is independent of mitophagy**, **related to** **Fig. 3.** **a** Detection of mitophagy using TOM20 and LC3B IF in the intestinal crypts of WT/WT*, WT/Mut** and Mut/Mut*** mice at 3, 8 and 12 months of age (scale bar, 10 μm). The percentage of crypts showing colocalization of TOM20 with LC3B is quantified below (Data are presented as the mean ± S.D and n=3 mice per group: two-way ANOVA test). **b** Detection of mitophagy using TOM20 and LC3B IF in the intestinal crypts of Mut/Mut*** mice at 8 months of age treated with NMN or water control (Scale bar, 10 μm). The percentage of crypts showing co-localization of TOM20 with LC3B is quantified below (Data are presented as the mean ± S.D and n=3 mice per group; N.S means no significance; unpaired two-tailed Student’s t test). **c** Number of mtDNA point mutations in the intestine of Mut/Mut*** mice at 8 months of age with NMN or water control (Data are presented as the mean ± S.D and n=3 mice per group; N.S, unpaired two-tailed Student’s t test). Source data are provided with this paper.

**
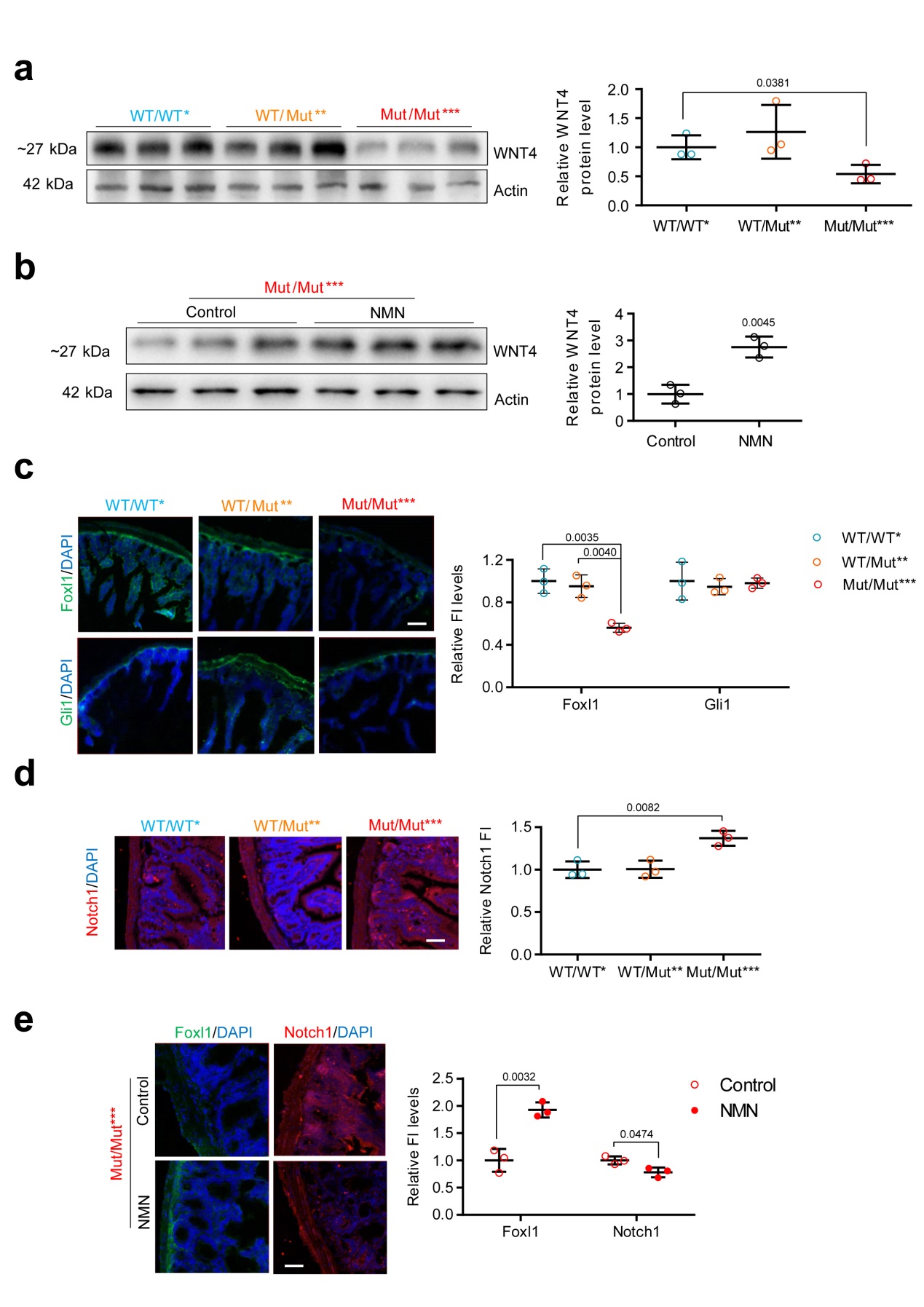
Supplementary Fig. 7 Increased mtDNA mutation burden induces downregulation of Foxl1 and upregulation of Notch1, related to** **Fig. 5**. **a** Protein expression of WNT4 by western blot analysis in the intestinal crypts of WT/WT*, WT/Mut** and Mut/Mut*** mice at 8 months. Relative band densities quantified using ImageJ are shown on right (Data are presented as the mean ± S.D and n=3 mice per group; one-way ANOVA test). **b** Protein expression of WNT4 by western blot analysis in the intestinal crypts of Mut/Mut*** mice at 8 months of age treated with NMN or water. Relative band densities quantified using ImageJ are shown on right (Data are presented as the mean ± S.D and n=3 mice per group; unpaired two-tailed Student’s t test). **c** Anti-Foxl1 and anti-Gli1 IF in the intestine of WT/WT*, WT/Mut** and Mut/Mut*** mice at 8 months. Relative FI of Foxl1 is quantified on right (Scale bar, 100 μm; Data are presented as the mean ± S.D and n=3 mice per group; one-way ANOVA test). **d** Anti-Notch1 IF in the intestine of WT/WT*, WT/Mut** and Mut/Mut*** mice at 8 months. Relative FI of Notch1 is quantified on right (Scale bar, 100 μm; Data are presented as the mean ± S.D and n=3 mice per group; one-way ANOVA test). **e** Anti-Foxl1 and anti-Notch1 IF in the intestine of Mut/Mut*** mice at 8 months of age with NMN or water control. Relative FI of Foxl1 and Notch1 is quantified on right (Data are presented as the mean ± S.D and n=3 mice per group; unpaired two-tailed Student’s t test). Source data are provided with this paper.

**
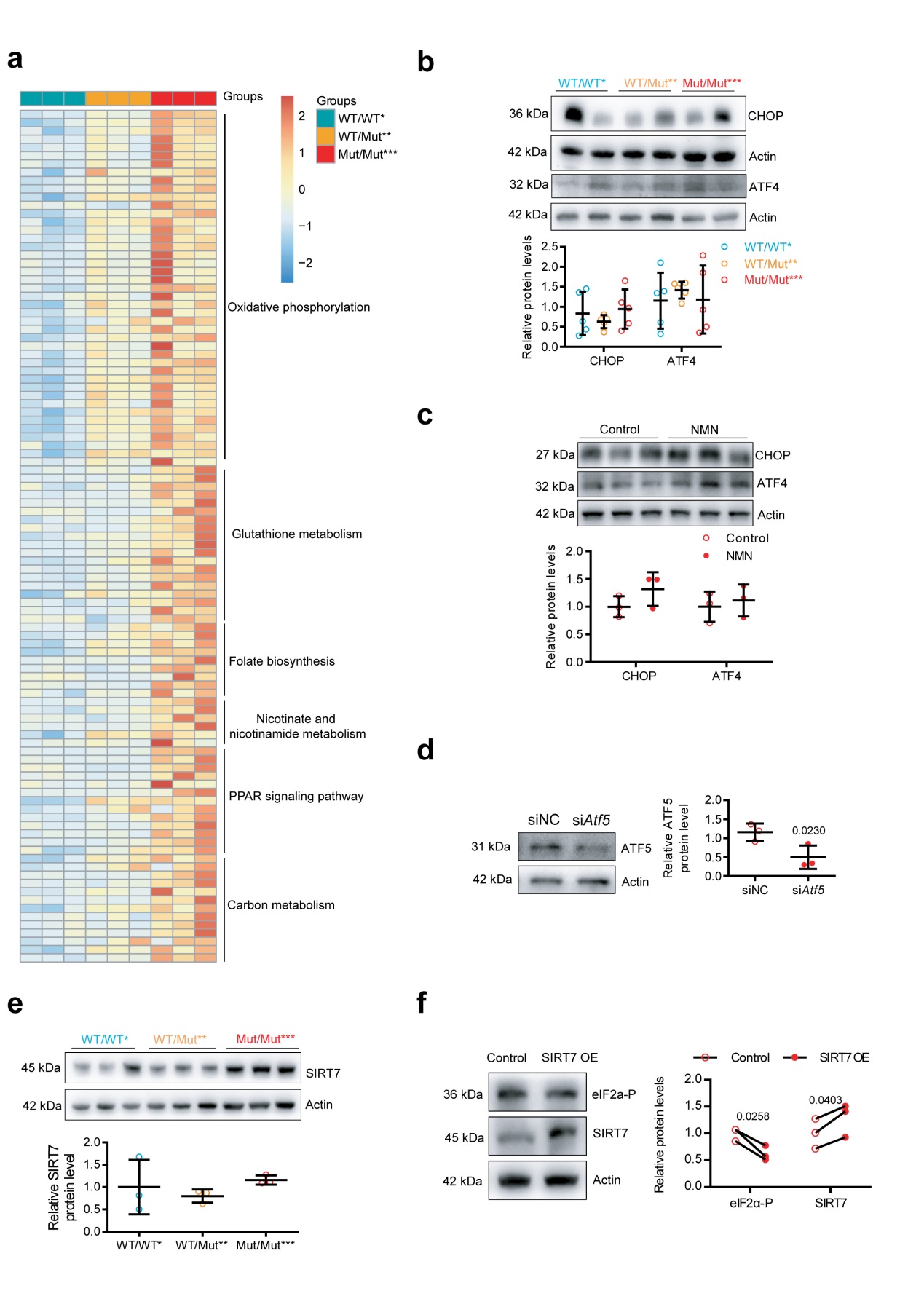
**

**Supplementary Fig. 8 Increased mtDNA mutation burden activates ISR, related to** **Fig. 6.** **a** Heatmap of differentially upregulated ISR genes in WT/WT*, WT/Mut** and Mut/Mut*** mice at 8 months (n=3 mice per group). **b** Protein expression of CHOP and ATF4 by western blot analysis in the intestinal crypts of WT/WT*, WT/Mut** and Mut/Mut*** mice at 8 months. Relative band densities quantified using ImageJ are shown at bottom (Data are presented as the mean ± S.D and n=5 mice per group). **c** Protein expression of CHOP and ATF4 by western blot analysis in the intestinal crypts of Mut/Mut*** mice treated with NMN or water control. Relative band densities quantified using ImageJ are shown at bottom (Data are presented as the mean ± S.D and n=3 mice per group; one-way ANOVA test). **d** Protein expression of ATF5 by western blot analysis in Mut/Mut*** intestinal crypt cells treated with siNC or si*Atf5*. Relative band densities quantified using ImageJ are shown on right (Data are presented as the mean ± S.D and n=3 mice per group; paired two-tailed Student’s t test). **e** Protein expression of SIRT7 by western blot analysis in the intestinal crypts of WT/WT*, WT/Mut** and Mut/Mut*** mice at 8 months. Relative band densities quantified using ImageJ are shown at bottom (Data are presented as the mean ± S.D and n=3 mice per group). **f** Protein expression of SIRT7 and eIF2α-P by western blot analysis in Mut/Mut*** intestinal crypt cells overexpressing (OE) SIRT7 or negative control. Relative band densities quantified using ImageJ are shown on right (Data are presented as the mean ± S.D and n=3 mice per group; paired two-tailed Student’s t test). Source data are provided with this paper.

**
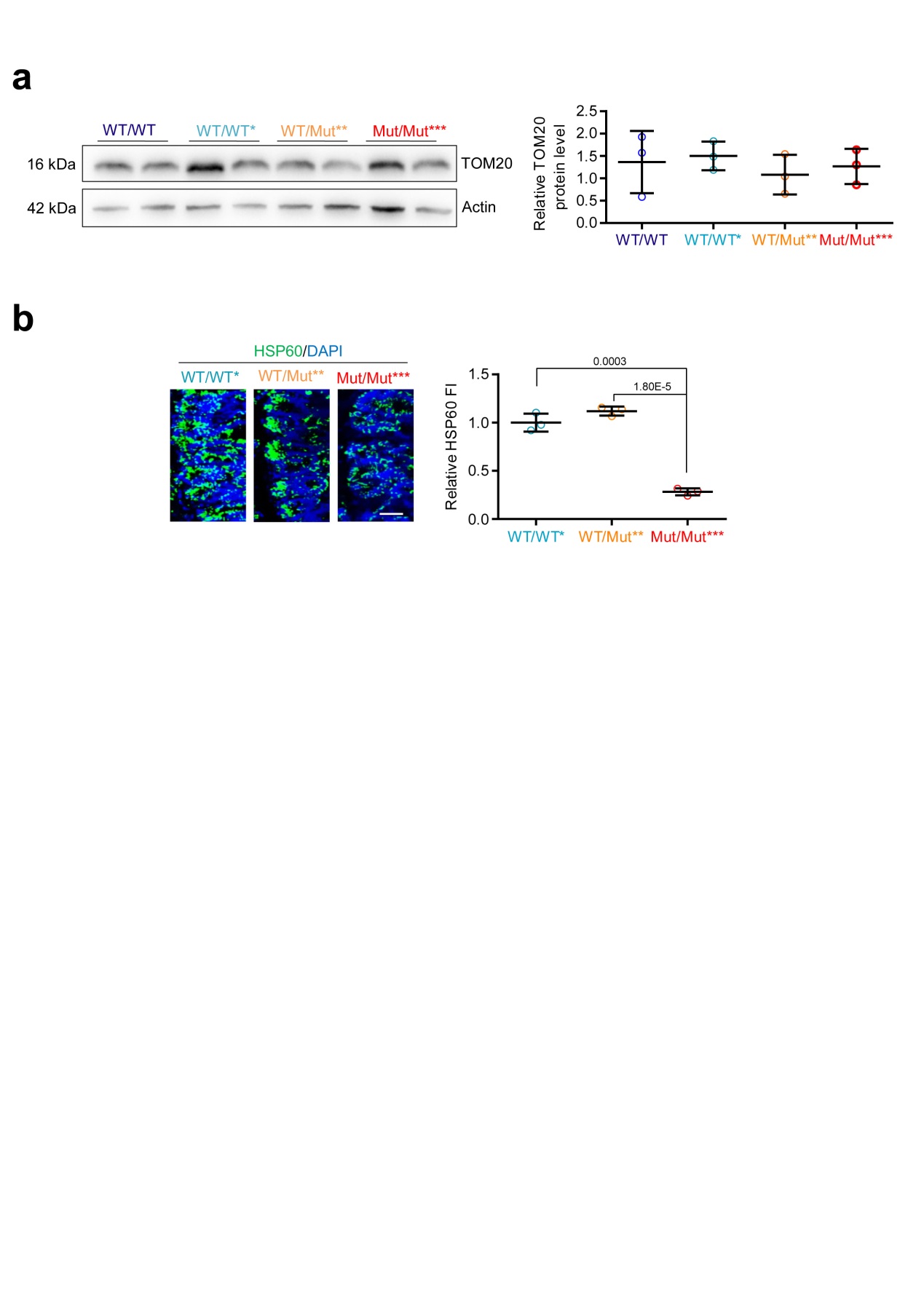
**

**Supplementary Fig. 9** **Anti-TOM20 western blotting and anti-HSP60 IF, related to Fig. 7. a** Western blot analysis of TOM20 and actin in the small intestine of WT/WT, WT/WT*, WT/Mut** and Mut/Mut*** mice at 8 months. Band densities were quantified using ImageJ, and relative values are shown on right (Data are presented as the mean ± S.D and n=3 mice per group). **b** Anti-HSP60 IF in the intestinal crypts in WT/WT*, WT/Mut** and Mut/Mut*** mice at 8 months (Scale bar, 20 μm). Relative HSP60 FI is quantified on right (Data are presented as the mean ± S.D and n=3 mice per group; one-way ANOVA test). Source data are provided with this paper.


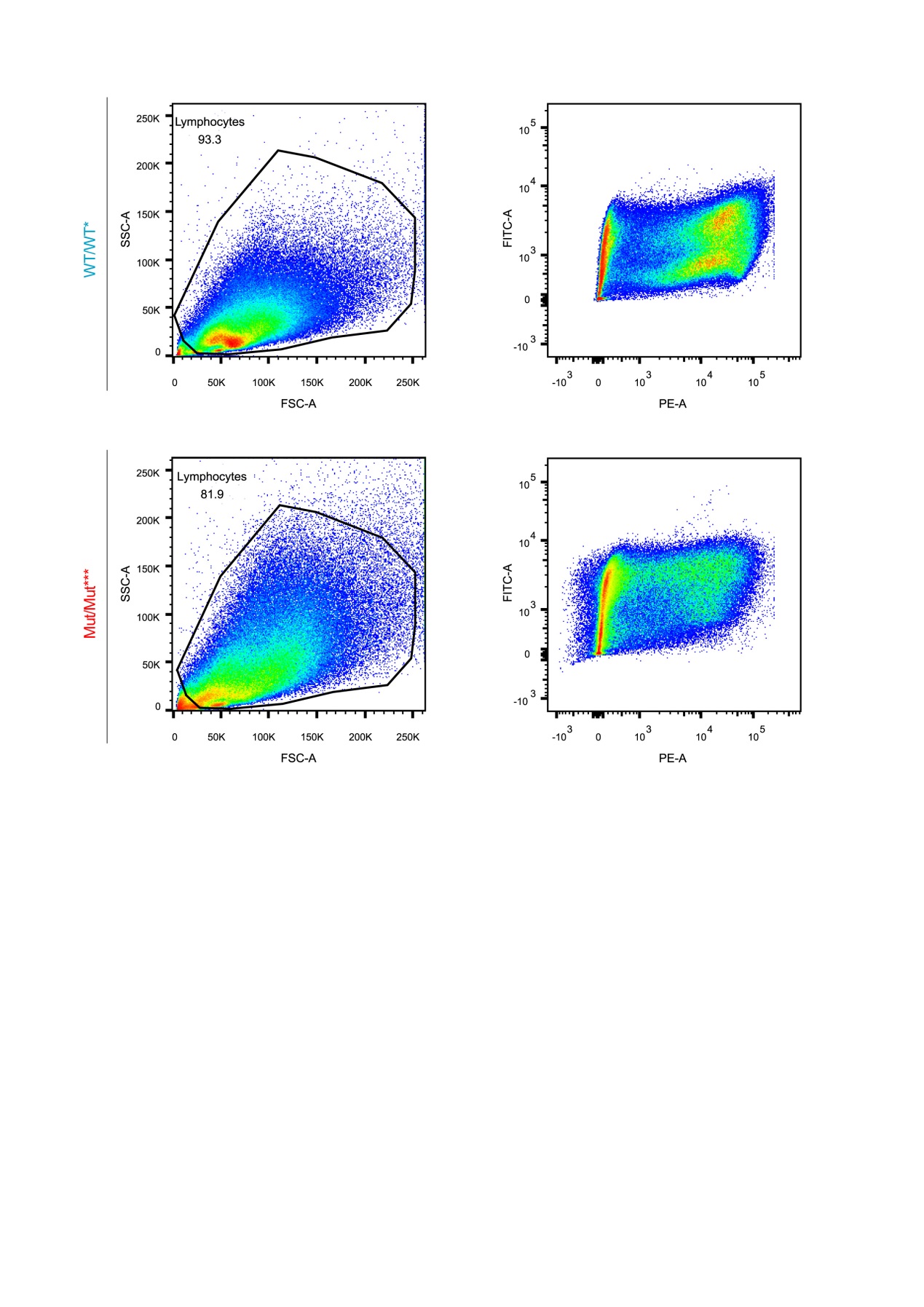


**Supplementary Fig. 10 Gating strategy**

Representative gating strategy to evaluate mitochondrial inner membrane potential, measured as JC-1 fluorescence by flow cytometry. Cell population was distinguished from cellular debris (left panel). Singlets were selected to gate JC-1 positive intestinal crypt cells (right panel). This gating strategy corresponds to supplementary figure 4c.
