# Supplementary material for: NAD+ dependent UPRmt activation underlies intestinal aging caused by mitochondrial DNA mutations
Source: Nat Commun. 2024 Jan 16;15:546. doi: 10.1038/s41467-024-44808-z (PMC10791663; doi:10.1038/s41467-024-44808-z)
Supplement: Supplementary file 3 — Reporting Summary [file 41467_2024_44808_MOESM3_ESM.pdf]

nature portfolio

Corresponding author(s): Xingguo Liu

Last updated by author(s): Dec 20, 2023

Reporting Summary

Nature Portfolio wishes to improve the reproducibility of the work that we publish. This form provides structure for consistency and transparency in reporting. For further information on Nature Portfolio policies, see our Editorial Policies and the Editorial Policy Checklist.

Statistics

For all statistical analyses, confirm that the following items are present in the figure legend, table legend, main text, or Methods section.

n/a	Confirmed	
☐	☒	The exact sample size (*n*) for each experimental group/condition, given as a discrete number and unit of measurement
☐	☒	A statement on whether measurements were taken from distinct samples or whether the same sample was measured repeatedly
☐	☒	The statistical test(s) used AND whether they are one- or two-sided<br>*Only common tests should be described solely by name; describe more complex techniques in the Methods section.*
☒	☐	A description of all covariates tested
☒	☐	A description of any assumptions or corrections, such as tests of normality and adjustment for multiple comparisons
☐	☒	A full description of the statistical parameters including central tendency (e.g. means) or other basic estimates (e.g. regression coefficient) AND variation (e.g. standard deviation) or associated estimates of uncertainty (e.g. confidence intervals)
☐	☒	For null hypothesis testing, the test statistic (e.g. F, t, r) with confidence intervals, effect sizes, degrees of freedom and P value noted<br>*Give P values as exact values whenever suitable.*
☒	☐	For Bayesian analysis, information on the choice of priors and Markov chain Monte Carlo settings
☒	☐	For hierarchical and complex designs, identification of the appropriate level for tests and full reporting of outcomes
☒	☐	Estimates of effect sizes (e.g. Cohen's d, Pearson's r), indicating how they were calculated

Our web collection on statistics for biologists contains articles on many of the points above.

Software and code

Policy information about availability of computer code

Data collection	BD Fortessa flow cytometer (BD Biosciences, USA) were used to collect data of flow cytometer.<br>Zeiss LSM 710, Zeiss LSM 880 and LSM 900 was used to collect image data.<br>RNA-sequencing reads were generated at illlumina NovaSeq 6000 platform (HeQin Bio-Technology Co..Ltd .Guangzhou, China).<br>Western blot signals were detected using a Mini Chemi910 Chemiluminescent/ Fluorescent Imaging and Analysis System(SageCreation, Beijing, China).
Data analysis	FlowJo V10 software were used to analyze data of flow cytometer;<br>RNA-seq data were analyzed using DESeq2 package of R software;<br>GraphPad 7 was used for Statistical analysis.<br>ImageJ 1.52a were used to analyze western blot bands density.

For manuscripts utilizing custom algorithms or software that are central to the research but not yet described in published literature, software must be made available to editors and reviewers. We strongly encourage code deposition in a community repository (e.g. GitHub). See the Nature Portfolio guidelines for submitting code & software for further information.

Data

Policy information about availability of data

All manuscripts must include a data availability statement. This statement should provide the following information, where applicable:

- Accession codes, unique identifiers, or web links for publicly available datasets
- A description of any restrictions on data availability
- For clinical datasets or third party data, please ensure that the statement adheres to our policy

The data and materials that support the findings of this study are available from the corresponding author (X.L.), upon reasonable request. The RNA-seq and mtDNA-seq data have been deposited in the Genome Sequence Archive (GSA) at the Beijing Institute of Genomics (BIG) Data Center, BIG, Chinese Academy of Sciences (https://bigd.big.ac.cn/gsa; RNA-seq accession number: CRA012233; mtDNA-seq accession number: CRA012237). The liquid chromatography-tandem mass spectrometry (LC-MS/MS) data for measuring NAD+ content have been deposited in the OMIX, China National Center for Bioinformation/Beijing Institute of Genomics, Chinese Academy of Sciences (https://ngdc.cncb.ac.cn/omix: accession no. OMIX004772). All other relevant data supporting the key findings of this study are available within the article and its supplementary information files. Any additional information is available upon request to the corresponding author (Xingguo Liu, liu_xingguo@gibh.ac.cn). Source data are provided with this paper.

Research involving human participants, their data, or biological material

Policy information about studies with human participants or human data. See also policy information about sex, gender (identity/presentation), and sexual orientation and race, ethnicity and racism.

Reporting on sex and gender	This study has no human participants.
Reporting on race, ethnicity, or other socially relevant groupings	This study has no human participants.
Population characteristics	This study has no human participants.
Recruitment	This study has no human participants.
Ethics oversight	This study has no human participants.

Note that full information on the approval of the study protocol must also be provided in the manuscript.

Field-specific reporting

Please select the one below that is the best fit for your research. If you are not sure, read the appropriate sections before making your selection.

☒ Life sciences ☐ Behavioural & social sciences ☐ Ecological, evolutionary & environmental sciences

For a reference copy of the document with all sections, see nature.com/documents/nr-reporting-summary-flat.pdf

Life sciences study design

All studies must disclose on these points even when the disclosure is negative.

Sample size	Sample size was estimated from preliminary experiments or from our previously published studies. No statistical method was applied to predetermine sample size. The FACS, Western blot and Immunofluorescence were conducted with at least three independentexperiments except otherwise stated. RNA-seq and mtDNA-seq were conducted with two or three independent samples. Animal experiments have been performed at least three times independently. Please Refer to figure legends and methods for details. All replications were successful.
Data exclusions	No data were excluded.
Replication	All mtDNA and RNA sequencing includes at east three independent biological replicates: all other experiments includes at east two biological replicates. We have added detailed description of replication in each experiment in Figure legends or Methods. The data exclusion criteria is described above.
Randomization	For measuring SoNar and FiNad ratio, the intestinal crypts were picked up at random. No randomization methods were utilized for other experiments as all samples are randomly collected from the population. Sample collections were performed with controls, all replications were successful and no data were excluded.
Blinding	The investigators were not blinded to group allocation during data collection and/or analysis. There is no need of blinding because most of experiments have been done by 2 or 3 researchers independently and are highly reproducible, besides, the RNA-seq results can cover whole transcriptome and do not cause any bias.

Reporting for specific materials, systems and methods

We require information from authors about some types of materials, experimental systems and methods used in many studies. Here, indicate whether each material, system or method listed is relevant to your study. If you are not sure if a list item applies to your research, read the appropriate section before selecting a response.

Materials & experimental systems

n/a	Involved in the study
	[x] Antibodies
[x]	[ ] Eukaryotic cell lines
[x]	[ ] Palaeontology and archaeology
	[x] Animals and other organisms
[x]	[ ] Clinical data
[x]	[ ] Dual use research of concern
[x]	[ ] Plants

Methods

n/a	Involved in the study
[x]	[ ] ChIP-seq
	[x] Flow cytometry
[x]	[ ] MRI-based neuroimaging

Antibodies

Antibodies used

anti-HSP60 (Abcam, ab46798, 1:1000 dilution for wb, 1:100 dilution for IF),
anti-LONP1 (Cell Signal Technology, 28020, 1:1000 dilution for wb),
anti-CDKN1A/p21 (Abclonal, A2691, 1:1000 dilution for wb),
anti-Phospho-eIF2α-S51 (Abclonal, AP0692, 1:1000 dilution for wb),
anti-ClpP (Abcam, ab124822, 1:1000 dilution for wb),
anti-ATF4 (Abcam, ab216839, 1:1000 dilution for wb),
anti-ATF5(Abcam, ab184923, 1:1000 dilution for wb),
anti-CHOP(Cell Signal Technology, 2895S, 1:1000 dilution for wb),
anti-TOM20 (Proteintech, 11802-1-AP, 1:1000 dilution for wb, 1:100 dilution for IF),
anti-WNT4 (Bio-Techne, MAB4751, 1:1000 dilution for wb, 1:100 dilution for IF),
anti-SIRT7 (Abclonal, A22735,1:1000 dilution for wb),
anti-WNT2 (Abclonal, A23997, 1:100 dilution for IF),
anti-WNT5A (Abclonal, A12744, 1:100 dilution for IF),
anti-Gli1 (Santa Cruz Biotechnology, sc-515751, 1:100 dilution for IF),
anti-Foxl1 (Santa Cruz Biotechnology, SC-130373, 1:100 dilution for IF),
anti-Actin (HUABIO, ET1702-67,1:1000 dilution for wb),
anti-β-catenin (Cell Signal Technology, 9562S,1:1000 dilution for wb, 1:100 dilution for IF),
HRP Conjugated Goat anti-Mouse IgG (HUABIO, HA1006, 1:3000 dilution for wb),
HRP Conjugated Goat anti-Rabbit IgG (HUABIO, HA1001, 1:3000 dilution for wb),
anti-Cyclin D1 (Abcam, ab16663, 1:100 dilution for IF),
anti-LC3B (Cell Signal Technology, 2775, 1:100 dilution for IF),
anti-Notch1 (Abclonal, A7636, 1:100 dilution for IF),
anti-Mouse IgG (H+L), Alexa Fluor 488 (Thermo Fisher Scientific, A-11001, 1:500 dilution for IF),
anti-Rabbit IgG (H+L), Alexa Fluor 488 (Thermo Fisher Scientific, A-11008, 1:500 dilution for IF)
anti-Rabbit IgG (H+L), Alexa Fluor 568, (Thermo Fisher Scientific, A-11011, 1:500 dilution for IF).

Validation

All the antibodies used in the manuscript were bought from commercial companies and are widely used for similar experiments by other researchers worldwide. The utility of these antibodies is stated on the websites of the corresponding suppliers. Antibody validation for Western blot analyses involved confirmation that the band corresponded to the reported molecular mass following gel migration. Antibody validation for Immunofluorescence analyses involved confirmation that corresponded to the reported protein location. Antibodies involved confirmation by the below web site are as follows:
anti-HSP60 (Abcam, ab46798, https://www.abcam.cn/products/primary-antibodies/hsp60-antibody-ab46798.html),
anti-LONP1 (Cell Signal Technology, 28020, https://www.cellsignal.cn/products/primary-antibodies/prss15-lonp1-d8w1j-rabbit-mab/28020),
anti-CDKN1A/p21 (Abclonal, A2691, https://abclonal.com.cn/catalog/A2691),
anti-Phospho-eIF2α-S51 (Abclonal, AP0692, https://abclonal.com.cn/catalog/AP0692),
anti-ClpP (Abcam, ab124822, https://www.abcam.cn/products/primary-antibodies/clpp-antibody-epr7133-ab124822.html),
anti-ATF4 (Abcam, ab216839, https://www.abcam.cn/products/primary-antibodies/atf-4-antibody-ab216839.html),
anti-ATF5(Abcam, ab184923, https://www.abcam.cn/products/primary-antibodies/atf5-antibody-epr18286-ab184923.html),
anti-CHOP(Cell Signal Technology, 2895S, https://www.cellsignal.cn/products/primary-antibodies/chop-l63f7-mouse-mab/2895),
anti-TOM20 (Proteintech, 11802-1-AP, https://www.ptgcn.com/products/TOM20-Antibody-11802-1-AP.htm),
anti-WNT4 (Bio-Techne, MAB4751, https://www.bio-techne.com/p/antibodies/human-mouse-wnt-4-antibody-55025_mab4751),
anti-SIRT7 (Abclonal, A22735, https://abclonal.com.cn/catalog/A22735),
anti-WNT2 (Abclonal, A23997, https://abclonal.com.cn/catalog/A23997),
anti-WNT5A (Abclonal, A12744, https://abclonal.com.cn/catalog/A12744),
anti-Gli1 (Santa Cruz Biotechnology, sc-515751, https://www.scbt.com/p/gli-1-antibody-c-1?requestFrom=search),
anti-Foxl1 (Santa Cruz Biotechnology, SC-130373, https://www.scbt.com/p/foxl1-antibody-40-m?requestFrom=search),
anti-Actin (HUABIO, ET1702-67,https://www.huabio.com/products/beta-actin-hrp-conjugated-antibody-clone-jf53-10-recombinant-monoclonal-et1702-67),
anti-β-catenin (Cell Signal Technology, 9562S,https://www.cellsignal.cn/products/primary-antibodies/b-catenin-antibody/9562),

anti-Cyclin D1 (Abcam, ab16663, https://www.abcam.cn/products/primary-antibodies/cyclin-d1-antibody-sp4-ab16663.html), anti-LC3B (Cell Signal Technology, 2775, https://www.cellsignal.cn/products/primary-antibodies/lc3b-antibody/2775), anti-Notch1 (Abclonal, A7636, https://abclonal.com.cn/catalog/A7636).

Animals and other research organisms

Policy information about studies involving animals; ARRIVE guidelines recommended for reporting animal research, and Sex and Gender in Research

Laboratory animals	PolgAD257A heterozygous mice (PolgAWT/Mut, Stock No: 017341, USA) and LGR5-EGFP-IRES-creERT2 heterozygous mice (LGR5WT/GFP Stock No: 008875, USA) were purchased from the Jackson Laboratory. Wild-type C57BL/6J mice with different ages (3 months, 8, 12 and 20 months) were purchased from the GemPharmatech co. Ltd (Nanjing, China). The male and female PolgAWT/Mut siblings were crossed to generate three genotype mice including WT/WT*, WT/Mut** and Mut/Mut*** with 3, 8 and 12 months of age. Mice were housed in an environment of suitable temperature (25°C) and humidity (typically 50%) under a 12h: 12h light/dark cycle (7 am/7 pm) with accessing to food and water ad libitum.
Wild animals	The study did not involve wild animals.
Reporting on sex	male mouse
Field-collected samples	The study did not involve samples collected from the field.
Ethics oversight	The same batch of male mice was used to perform experiments in accordance with relevant guidelines and regulations, which had been reviewed and approved by Guangzhou Institutes of Biomedicine and Health Ethical Committee (Approve no. 2018040).

Note that full information on the approval of the study protocol must also be provided in the manuscript.

Flow Cytometry

Plots

Confirm that:

- [x] The axis labels state the marker and fluorochrome used (e.g. CD4-FITC).
- [x] The axis scales are clearly visible. Include numbers along axes only for bottom left plot of group (a 'group' is an analysis of identical markers).
- [x] All plots are contour plots with outliers or pseudocolor plots.
- [x] A numerical value for number of cells or percentage (with statistics) is provided.

Methodology

Sample preparation	Primary cells of mouse small intestinal crypts were isolated and labeled according to the kit instructions.
Instrument	BD Fortessa flow cytometer (BD Biosciences, USA)
Software	FlowJo V10 software
Cell population abundance	At least 100,000 cells were collected when analyzing cell samples
Gating strategy	The gating strategies used in this study haves been provided in supplementary files. The positive and negative populations are defined according to the expression levels (fluorescent intensity) of markers in the analysis.

- [x] Tick this box to confirm that a figure exemplifying the gating strategy is provided in the Supplementary Information.
